# Supplementary figures and images for: A laboratory comparison of the interactions between three plastic mulch types and 38 active substances found in pesticides
Source: PeerJ. 2020 Sep 21;8:e9876. doi: 10.7717/peerj.9876 (PMC7513747; doi:10.7717/peerj.9876)

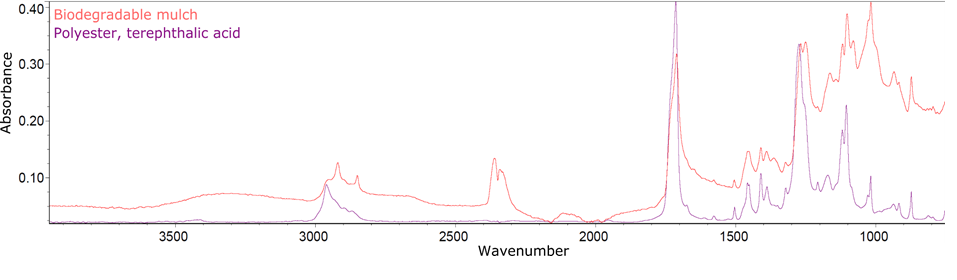

Supplement: Supplemental Information 1 [file peerj-08-9876-s001.png]

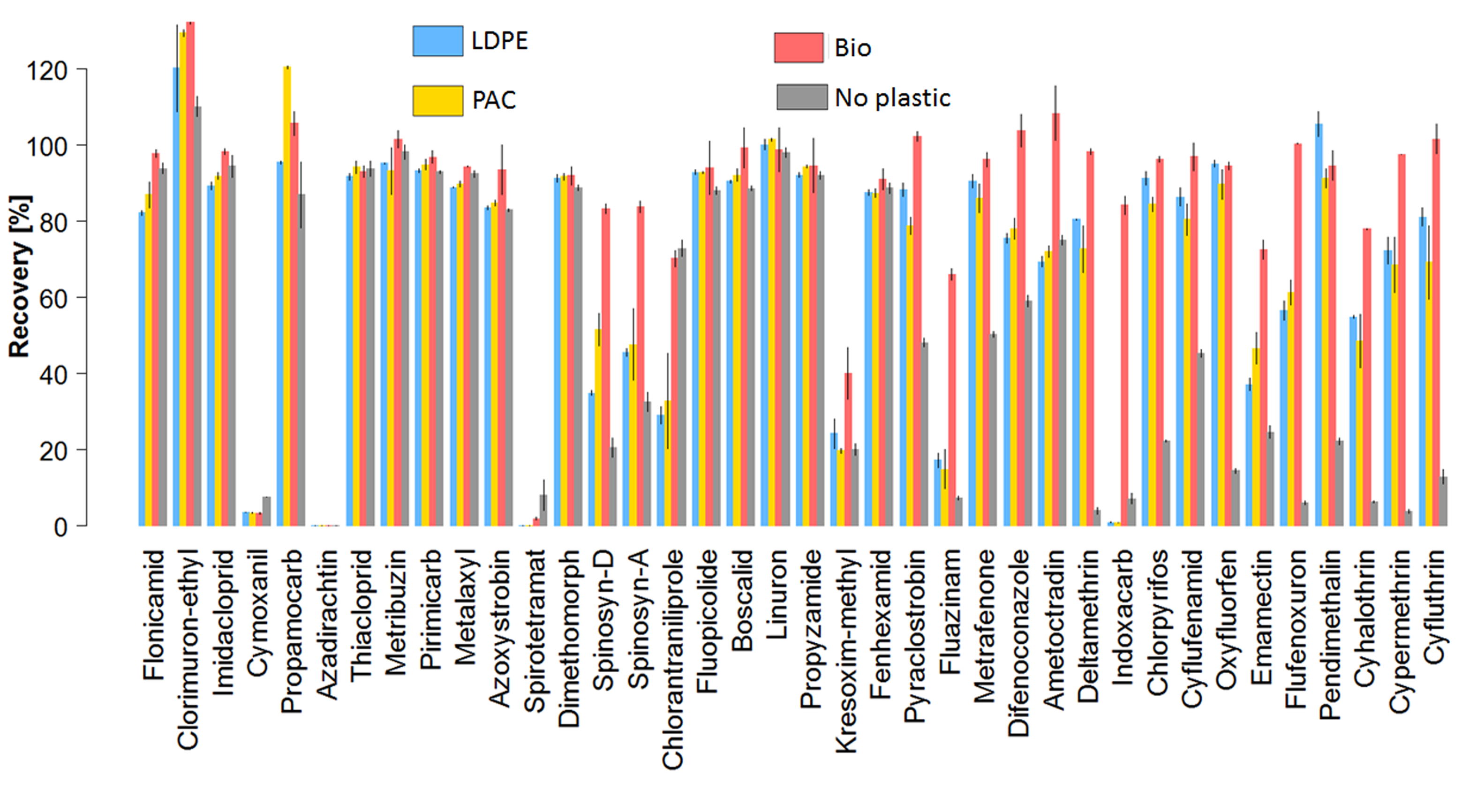

Supplement: Supplemental Information 2 — Black lines at the top of each column represent the measurement ranges (min and max). Active substances are ordered according to increasing log P (octanol-water partition coefficient) from left to right. [file peerj-08-9876-s002.png]
