# Supplementary material for: A laboratory comparison of the interactions between three plastic mulch types and 38 active substances found in pesticides
Source: PeerJ. 2020 Sep 21;8:e9876. doi: 10.7717/peerj.9876 (PMC7513747; doi:10.7717/peerj.9876)
Supplement: Supplemental Information 3 — The comparisons were made with the references spectra of the high resolution libraries HR Hummel Polymer and Additives, HR Spectra Polymers and Plasticizers by ATR and HR Sprouse Polymers by Transmission. [file peerj-08-9876-s003.docx]

| **Match [%]** | **Compound Name** | **Library Name** |
| --- | --- | --- |
| 78.32 | Polyester, terephthalic acid | HR Hummel Polymer and Additives |
| 72.77 | Poly(butylene terephthalate) | HR Specta Polymers and Plasticizers by ATR - corrected |
| 72.29 | Polyester, tere- & isophthalic acids | HR Hummel Polymer and Additives |
| 71.3 | Poly(1,4-butylene terephthalate) | HR Hummel Polymer and Additives |
| 70.7 | Poly(1,4-butylene terephthalate) | HR Hummel Polymer and Additives |
| 70.52 | Poly(butylene terephthalate) | HR Spectra Polymers and Plasticizers by ATR |
| 68.37 | Polyester, tere- & isophthalic acids | HR Hummel Polymer and Additives |
| 68.11 | Polyester, tere- & isophthalic acids | HR Hummel Polymer and Additives |
| 65.71 | Poly(1,4-butylene terephthalate) | HR Sprouse Polymers by Transmission |
| 65.6 | Poly(ethylene terephthalate) | HR Specta Polymers and Plasticizers by ATR - corrected |
