# Supplementary material for: A laboratory comparison of the interactions between three plastic mulch types and 38 active substances found in pesticides
Source: PeerJ. 2020 Sep 21;8:e9876. doi: 10.7717/peerj.9876 (PMC7513747; doi:10.7717/peerj.9876)
Supplement: Supplemental Information 4 — A mobile phases of 0.1% formic acid and 0.5% ammonium formate in water (eluent A) or in 95% methanol, 5% water (eluent B) was used for the LC-MS/MS analysis. [file peerj-08-9876-s004.docx]

| **Time [min]** | **Eluent A [%]** | **Eluent B [%]** |
| --- | --- | --- |
| 0 | 100 | 0 |
| 2.5 | 55 | 45 |
| 8 | 0 | 100 |
| 11 | 0 | 100 |
| 12 | 100 | 0 |
| 14 | 100 | 0 |
