# Supplementary material for: A laboratory comparison of the interactions between three plastic mulch types and 38 active substances found in pesticides
Source: PeerJ. 2020 Sep 21;8:e9876. doi: 10.7717/peerj.9876 (PMC7513747; doi:10.7717/peerj.9876)
Supplement: Supplemental Information 5 — The molar mass, solubility in water at 20 °C, aqueous hydrolysis half-life time at 20 °C and pH 7 (DT50 in water) and octanol-water partition coefficient at pH 7, 20 °C (log P) were obtained from the Pesticide Properties DataBase (PPDB, 2018). [file peerj-08-9876-s005.docx]

| **Compound** | **Category** | **Molar mass [g/mol]** | **Solubility [mg/L]** | **DT50 in water  [days]** | **log P [-]** |
| --- | --- | --- | --- | --- | --- |
| Ametoctradin | fungicide | 275.39 | 0.15 | 1.8 | 4.4 |
| Azadirachtin | insecticide | 720.721 | 260 | 8 | 1.09 |
| Azoxystrobin | fungicide | 403.4 | 6.7 | 84.5 | 2.5 |
| Boscalid | fungicide | 343.21 | 4.6 | 484.4 | 2.96 |
| Chlorantraniliprole | insecticide | 483.15 | 3446 | 597 | 2.86 |
| Chlorpyrifos | insecticide | 350.58 | 1.05 | 386 | 4.7 |
| Clorimuron-ethyl | herbicide | 414.82 | 1200 | 40 | 2.5 |
| Cyflufenamid | fungicide | 412.36 | 0.52 | 210 | 4.7 |
| Cyfluthrin | insecticide | 434.29 | 0.0066 | 51 | 6 |
| Lambda-cyhalothrin | insecticide | 449.85 | 0.005 | 57 | 5.5 |
| Cymoxanil | fungicide | 198.18 | 780 | 1.4 | 0.67 |
| Cypermethrin | insecticide | 416.3 | 0.004 | 70 | 5.55 |
| Deltamethrin | insecticide | 505.2 | 0.0002 | 26 | 4.6 |
| Difenoconazole | fungicide | 406.26 | 15 | 130 | 4.36 |
| Dimethomorph | fungicide | 387.86 | 28.95 | 56.7 | 2.68 |
| Emamectin | insecticide | 886.133 | 24 | - | 5 |
| Fenhexamid | fungicide | 302.2 | 24 | 0.43 | 3.51 |
| Flonicamid | insecticide | 229.16 | 5200 | 1.1 | -0.24 |
| Fluazinam | fungicide | 465.14 | 0.135 | 124 | 4.87 |
| Flufenoxuron | insecticide | 488.77 | 0.0043 | 72.5 | 5.11 |
| Fluopicolide | fungicide | 383.58 | 2.8 | 271 | 2.9 |
| Imidacloprid | insecticide | 255.66 | 610 | 187 | 0.57 |
| Indoxacarb | insecticide | 527.83 | 0.2 | 113.2 | 4.65 |
| Kresoxim-methyl | fungicide | 313.35 | 2 | 0.87 | 3.4 |
| Linuron | herbicide | 249.09 | 63.8 | 57.6 | 3 |
| Metalaxyl | fungicide | 279.33 | 8400 | 36 | 1.75 |
| Metrafenone | fungicide | 409.3 | 0.492 | 250.6 | 4.3 |
| Metribuzin | herbicide | 214.29 | 10700 | 7.1 | 1.75 |
| Oxyfluorfen | herbicide | 361.7 | 0.116 | 138 | 4.86 |
| Pendimethalin | herbicide | 281.31 | 0.33 | 182.3 | 5.4 |
| Pirimicarb | insecticide | 238.39 | 3100 | 86 | 1.7 |
| Propamocarb | fungicide | 188.3 | 900000 | 14 | 0.84 |
| Propyzamide | herbicide | 256.13 | 9 | 50.5 | 3.27 |
| Pyraclostrobin | fungicide | 387.8 | 1.9 | 62 | 3.99 |
| Spinosyn-A | insecticide | 731.98 | 14.5 | 24.3 | 3.9 |
| Spinosyn-D | insecticide | 745.98 | 0.76 | 45.2 | 4.3 |
| Spirotetramat | insecticide | 373.48 | 29.9 | 0.19 | 2.51 |
| Thiacloprid | insecticide | 252.72 | 184 | 1.3 | 1.26 |
