# Supplementary material for: A laboratory comparison of the interactions between three plastic mulch types and 38 active substances found in pesticides
Source: PeerJ. 2020 Sep 21;8:e9876. doi: 10.7717/peerj.9876 (PMC7513747; doi:10.7717/peerj.9876)
Supplement: Supplemental Information 6 — LDPE: Low Density Polyethylene mulch; PAC: Pro-oxidant Additive Containing mulch; Bio: Biodegradable mulch. [file peerj-08-9876-s006.docx]

| **Active substance** | **LOQ [ng/**  **mL]** | **Sorption on LDPE [%]** | **Sorption on PAC [%]** | **Sorption on Bio [%]** | **Decay with LDPE [%]** | **Decay with PAC [%]** | **Decay with Bio [%]** | **Decay without plastic [%]** |
| --- | --- | --- | --- | --- | --- | --- | --- | --- |
| Ametoctradin | 2.5 | 8; 8.8 | 6.5; 7.2 | 59.9; 67.1 | 29.2; 32 | 26.4; 29.5 | -15.6; -1.2 | 23.7; 26.2 |
| Azadirachtin | 2.5 | Below LOQ | Below LOQ | Below LOQ | 100; 100 | 100; 100 | 100; 100 | 100; 100 |
| Azoxystrobin | 0.125 | 0.4; 0.4 | 0.4; 0.4 | 46.3; 52 | 16; 17 | 14.4; 15.9 | 0.1; 12.9 | 16.6; 17.4 |
| Boscalid | 0.125 | 5.7; 6 | 3.9; 4.4 | 69.6; 74.8 | 9.1; 10 | 6.2; 9.5 | -4.5; 6 | 10.8; 12 |
| Chlorantraniliprole | 0.125 | 0.1; 0.1 | 0.02; 0.04 | 17; 19.8 | 68.7; 73.3 | 54.6; 79.6 | 27.6; 31.9 | 25; 29.2 |
| Chlorpyrifos | 1 | 86.8; 90 | 79.8; 83.5 | 94.9; 96.2 | 7; 10.3 | 13.7; 17.4 | 2.9; 4.4 | 77.3; 77.9 |
| Clorimuron-ethyl | 0.125 | 0.01; 0.02 | 0.02; 0.02 | 0.2; 0.2 | -31; -8.9 | -30; -28.6 | -33; -31.7 | -12.9; -7.5 |
| Cyflufenamid | 0.125 | 44.1; 46.9 | 34.5; 39.1 | 91; 97.6 | 11.2; 16 | 15.3; 23.8 | -0.6; 6.7 | 53.6; 55.7 |
| Cyfluthrin | 2.5 | 78.4; 82.9 | 59.1; 78.3 | 97.2; 105 | 17.1; 21.6 | 21.7; 40.9 | -5; 2.8 | 85.3; 89 |
| Lembda-cyhalothrin | 2.5 | 54.6; 55.2 | 41.6; 55.7 | 77.9; 78 | 44.8; 45.4 | 44.4; 58.5 | 22; 22.1 | 93.5; 93.9 |
| Cymoxanil | 1 | Below LOQ | Below LOQ | Below LOQ | 96.4; 96.5 | 96.5; 96.8 | 96.5; 96.9 | 92.5; 92.5 |
| Cypermethrin | 2.5 | 68.8; 75.7 | 61.4; 75.8 | 97.5; 97.6 | 24.3; 31.2 | 24.2; 38.6 | 2.4; 2.5 | 95.8; 96.7 |
| Deltamethrin | 2.5 | 80.4; 80.6 | 66.6; 78.8 | 97.6; 98.9 | 19.4; 19.6 | 21.2; 33.4 | 1.1; 2.4 | 95.1; 96.9 |
| Difenoconazole | 0.125 | 26.8; 28.4 | 18; 21.1 | 92.8; 99.4 | 23.2; 25.7 | 19.1; 24.7 | -8.1; 0.4 | 39.6; 42.5 |
| Dimethomorph | 0.125 | 0.3; 0.4 | 0.3; 0.3 | 16.5; 19.1 | 7.7; 9.6 | 7.4; 9.1 | 5.8; 10.4 | 10.6; 11.9 |
| Emamectin | 0.125 | 16.8; 18.1 | 4.1; 4.3 | 64.9; 68.6 | 61.2; 64.5 | 49.2; 57.5 | 24.9; 30 | 73.8; 76.9 |
| Fenhexamid | 0.5 | 0.5; 0.5 | 0.5; 1 | 14.9; 16.9 | 11.6; 13.1 | 11.6; 13.6 | 6.3; 11.8 | 9.9; 12.7 |
| Flonicamid | 0.125 | Below LOQ | Below LOQ | 0.01; 0.02 | 17.2; 18.3 | 9.7; 16.4 | 1.2; 3.2 | 4.7; 7.5 |
| Fluazinam | 5 | 10.6; 13.5 | 5.8; 15.2 | 64.7; 67.5 | 81; 84.6 | 80.1; 90.3 | 32.5; 35.4 | 92.3; 93.1 |
| Flufenoxuron | 0.125 | 52.5; 57.2 | 56; 62.2 | 100; 100.5 | 41; 45.9 | 35.5; 42 | -0.6; -0.2 | 93.4; 94.5 |
| Fluopicolide | 0.125 | 3.5; 3.7 | 2.4; 2.7 | 52.4; 60.2 | 6.5; 7.8 | 6.9; 7.6 | -1.1; 13 | 10.8; 13.1 |
| Imidacloprid | 0.125 | Below LOQ | Below LOQ | 0.2; 0.2 | 9.7; 11.7 | 7.1; 9 | 1; 2.4 | 2.6; 8.4 |
| Indoxacarb | 0.125 | 0.4; 0.4 | 0.4; 0.4 | 81.1; 85.8 | 99.1; 99.2 | 99.2; 99.2 | 13.3; 18.3 | 91.4; 94.3 |
| Kresoxim-methyl | 5 | 14.8; 22 | 3.5; 4.7 | 33.3; 42.8 | 71.9; 79.8 | 79.6; 81.1 | 53.3; 66.7 | 78.4; 81.5 |
| Linuron | 2.5 | 3.4; 3.8 | 2.3; 2.6 | 44.3; 48.1 | -1.5; 1.2 | -1.9; -1.1 | -4.5; 7 | 0.6; 3.1 |
| Metalaxyl | 2.5 | Below LOQ | Below LOQ | 0.4; 0.4 | 11; 11.2 | 9.4; 11 | 5.5; 5.8 | 6.8; 8.3 |
| Metrafenone | 0.125 | 49.8; 52.8 | 44.6; 48.5 | 90.2; 92.9 | 7.8; 11.3 | 10.3; 17.8 | 1.9; 5.7 | 48.9; 50.6 |
| Metribuzin | 2.5 | Below LOQ | Below LOQ | 0.9; 1.3 | 4.6; 4.8 | 0.6; 13 | -3.8; 0.6 | -0.1; 3.7 |
| Oxyfluorfen | 0.25 | 89; 90.4 | 80.1; 86.7 | 93.3; 95 | 4.1; 5.6 | 6.5; 14.1 | 4.6; 6.4 | 84.9; 86.1 |
| Pendimethalin | 1 | 100; 106 | 86.2; 91.1 | 90.7; 98.6 | -8.9; -2.4 | 6.2; 11.2 | 1.4; 9.3 | 76.8; 78.7 |
| Pirimicarb | 0.125 | 0.1; 0.1 | 0.1; 0.1 | 0.3; 0.3 | 6.1; 7.2 | 3.7; 6.5 | 1.5; 4.8 | 6.7; 7.4 |
| Propamocarb | 0.25 | 0.3; 0.3 | Below LOQ | 0.3; 0.3 | 4.1; 4.9 | -20.8; -20 | -8.7; -2.7 | 4.4; 21.6 |
| Propyzamide | 2.5 | 3.6; 3.8 | 2.5; 2.8 | 41.2; 48.6 | 7.3; 8.6 | 5.2; 6 | -1.7; 12.5 | 7; 8.9 |
| Pyraclostrobin | 0.125 | 38; 40.2 | 29.9; 34 | 98.5; 100 | 10; 13.5 | 18.9; 23.4 | -3.7; -1 | 50.7; 53.1 |
| Spinosyn-A | 0.125 | 9.4; 9.9 | 9.8; 11.7 | 67; 67.2 | 53.5; 55.4 | 43.1; 61.6 | 14.7; 17.8 | 65; 69.9 |
| Spinosyn-D | 0.125 | 13.1; 13.7 | 15.3; 16.3 | 74.3; 75 | 64.3; 65.8 | 44.1; 52.8 | 15.4; 17.9 | 77; 81.9 |
| Spirotetramat | 0.125 | Below LOQ | Below LOQ | 0.1; 0.3 | 100; 100 | 100; 100 | 97.8; 98.6 | 87.9; 95.9 |
| Thiacloprid | 0.125 | 0.05; 0.05 | 0.03; 0.03 | 0.8; 1 | 7.4; 9.1 | 4.2; 7.4 | 5.5; 8.5 | 4.2; 8.2 |
